# Direct detection of resistance to fluoroquinolones/SLIDs in sputum specimen by GenoType MTBDR*sl* v.2.0 assay A study from Eastern Uttar Pradesh, India

**DOI:** 10.1186/s12941-021-00463-6

**Published:** 2021-08-26

**Authors:** Kamal Singh, Richa Kumari, Smita Gupta, Rajneesh Tripathi, Anjali Srivastava, Vidisha Shakya, Ankush Gupta, Shampa Anupurba

**Affiliations:** 1 Department of Microbiology, Institute of Medical Sciences, Banaras Hindu University, Varanasi, Uttar Pradesh India; 2 Department of Botany (Applied Microbiology), Institute of Science, Banaras Hindu University, Varanasi, Uttar Pradesh India; 3 Department of Biochemistry, Institute of Science, Banaras Hindu University, Varanasi, Uttar Pradesh India

**Keywords:** GenoType MTBDR*sl* v.2.0 assay, Fluoroquinolones (FQs), Second-line injectable drugs (SLIDs), RR-TB, MDR-TB, XDR-TB

## Abstract

**Background:**

According to World Health Organization (WHO), drug-resistant tuberculosis (DR-TB) is a major contributor to antimicrobial resistance globally and continues to be a public health threat. Annually, about half a million people fall ill with DR-TB globally. The gradual increase in resistance to fluoroquinolones (FQs) and second-line injectable drugs (SLIDs), poses a serious threat to effective TB control and adequate patient management. Therefore, WHO suggests the use of GenoType MTBDR*sl* v.2.0 assay for detection of multiple mutations associated with FQs and SLIDs. Hence, the study was conducted to determine the prevalence of resistance to FQs and SLIDs by comparing direct GenoType MTBDR*sl* v.2.0 assay with phenotypic drug susceptibility testing (DST).

**Methods:**

The study was conducted on 1320 smear positive sputum samples from a total of 2536 RR-TB, confirmed by GeneXpert MTB/RIF. The smear positive specimens were decontaminated, and DNA extraction was performed. Furthermore, the extracted DNA was used for GenoType MTBDR*sl* v.2.0 assay. While 20% of the decontaminated specimens were inoculated in Mycobacterium growth indicator tube (MGIT) for drug susceptibility testing (DST).

**Results:**

Out of 1320 smear positive sputum samples, 1178 were identified as *Mycobacterium tuberculosis* complex (MTBC) and remaining were negative by GenoType MTBDR*sl* v.2.0 assay. Of the 1178 MTBC positive, 26.6% were sensitive to both FQs and SLIDs, whereas 57.3% were only FQs resistant and 15.9% were resistant to both FQs and SLIDs. Further DST of 225 isolates by liquid culture showed that 17% were sensitive to both FQs and SLIDs, 61.3% were only FQs resistant and 21.3% were resistant to both. The specificity for FQs and SLIDs was 92.31% and 100% whereas sensitivity was 100% respectively by GenoType MTBDR*sl* v.2.0 assay in direct sputum samples.

**Conclusions:**

Our study clearly suggests that GenoType MTBDR*sl* v.2.0 assay is a reliable test for the rapid detection of resistance to second-line drugs after confirmation by GeneXpert MTB/RIF assay for RR-TB. Though, high rate FQ (ofloxacin) resistance was seen in our setting, moxifloxacin could be used as treatment option owing to very low resistance.

**Supplementary Information:**

The online version contains supplementary material available at 10.1186/s12941-021-00463-6.

## Background

Drug-resistant tuberculosis (DR-TB) has emerged as an enormous global challenge for TB control. It develops either because of an infection with a resistant strain or as a result of inadequate treatment. Multidrug-resistant TB (MDR-TB) is the form of TB that is resistant to rifampicin and isoniazid, the two most powerful anti-TB drugs [1]. It is of grave concern to public health, with higher mortality rates than drug-susceptible TB [2]. These TB patients require treatment with second-line drugs that are costlier and have increased toxicity; and remain infectious longer than patients infected with drug-susceptible strains [3]. Extensively drug-resistant TB (XDR-TB) is defined as MDR-TB plus resistance to fluoroquinolones (FQs) and one of the second line injectable drugs (SLIDs) utilized in MDR-TB treatment regimens [4].

With the increasing use of the GeneXpert MTB/RIF assay for simultaneous detection of TB and resistance to rifampicin (without further testing for isoniazid resistance), an increasing number of RR-TB cases are being detected and notified [5]. Rifampicin resistant TB (RR-TB) is considered as a surrogate marker for MDR-TB [6].

According to the WHO report 2019, there were an estimated 484 000 (range, 417 000–556 000) incident cases of MDR/RR-TB in 2018. Worldwide fifty percent cases of MDR/RR-TB were in India (27%), China (14%), and Russia (9%). In 2018, there were about 214 000 (range, 133 000–295 000) deaths from MDR/RR-TB. Among the MDR/RR-TB patients, 59% were notified as XDR-TB. The five countries that reported the foremost number of XDR-TB cases were Belarus, India, Russia, South Africa, and Ukraine [7].

In recent years several molecular methods have been developed for the diagnosis of tuberculosis and detection of drug resistance, including line probe assays [8]. Most of the species of *Mycobacteria* grow slowly with a generation time of 18 to 24 h; resulting in delay of TB diagnosis by conventional methods [9]. Molecular techniques reducing turnaround time (TAT) are able to detect and identify *Mycobacterium tuberculosis complex* (MTBC) within 1–2 days instead of several weeks with the traditional methods [10]. It is essential to detect drug resistance to second-line drugs before initiating treatment for better management of MDR-TB.

Line probe assays (LPAs) detect mutations associated with drug resistance. The Genotype MTBDR*plus* assay (Hain Lifescience Nehren Germany) identifies MTBC and detects resistance to rifampicin and isoniazid. GenoType MTBDR*sl* v.1.0 assay detects resistance to FQs, SLIDs and ethambutol (EMB) [11]. However, in recent years several novel mutations were identified that also confer resistance. Thus, WHO recommended the use of GenoType MTBDR*sl* v.2.0 assay (Hain Lifescience Nehren German) which detects multiple mutations which are associated with resistance to FQs and SLIDs. Mutations in *gyrA* and *gyrB* are detected for resistance to FQs, while resistance to SLIDs are detected through mutations in *rrs* and *eis* gene [12]. In 2018, Revised National Tuberculosis Control Program (RNTCP) recommended GenoType MTBDR*sl* v.2.0 as the initial test, instead of phenotypic drug susceptibility testing (DST) [13]. Uttar Pradesh is a region where highest number of TB cases are reported annually in India (https://tbfacts.org/tb-statistics-india/). With this in mind, the study was designed to compare the direct GenoType MTBDR*sl* v.2.0 assay with phenotypic drug susceptibility testing and determine the prevalence of resistance to FQs and SLIDs.

## Methods

### Location and samples

The study was undertaken in TB Culture & DST lab, Department of Microbiology, Institute of Medical Sciences, Banaras Hindu University, Varanasi, India, over the period of January 2019 to December 2019. Specimen processing, inoculation, and DST were performed in RNTCP certified laboratory with well-equipped BSL II & III facilities under standard biosafety precautions. All presumptive TB samples were directly detected by GeneXpert MTB/RIF assay, which identified TB along with rifampicin resistance (RR). Since RR is a surrogate marker for MDR-TB, the diagnosis of MDR-TB was based on this data. Over a period of one year, a total of 2536 RR-TB sputum specimens were collected, among which 1320 samples were smear positive and 1216 were smear negative. The smear positive samples were included in this study and subjected to GenoType MTBDR*sl* v.2.0 assay while phenotypic DST was performed for 20% of the samples. Every fifth sample was systematically selected for culture.

### Specimen processing and Genomic DNA extraction

The collected specimens were decontaminated as described elsewhere [6, 14]. Briefly, specimens were decontaminated using N-acetyl-L-cysteine and sodium hydroxide (NALC-NaOH) method. The decontaminated and concentrated sediments were used for Ziehl–Neelsen staining (ZN staining) as well as for DNA extraction and inoculation into BACTEC MGIT 960 (BD, USA) automated liquid culture system for DST.

DNA extraction was performed by using GenoLyse kit (Hain Lifescience, Nehren, Germany) according to manufacturer’s instructions. In brief, 1000 μl of the decontaminated sample was centrifuged at 10,000×*g* for 15 min. Supernatant was discarded and the pellet was re-suspended into 100 μl lysis buffer followed by vortexing for 30 s. This suspension was incubated in water bath at 95 ◦C for 5 min. Following this 100 μl neutralization buffer was added and vortexed for 30 s, then it was centrifuged at 13,000×*g* for 5 min. The supernatant containing DNA was transferred in a separate tube for further use.

### GenoType MTBDR*sl* v.2.0 assay

The extracted genomic DNA was subjected to GenoType MTBDR*sl* v.2.0 assay for the detection of MTBC and resistance to FQs and SLIDs, according to the manufacturer’s instructions (Hain Lifescience, Nehren, Germany).

### Drug susceptibility testing

For 264 samples a portion of the decontaminated samples (0.5 ml) was inoculated into a mycobacterium growth indicator tube (MGIT) and incubated in an automated BACTEC MGIT-960 system for a maximum of 42 days, with constant monitoring. ZN microscopy was performed from each positive-flagged MGIT tube to check for the presence of acid-fast bacilli (AFB). Simultaneously, a loopful of liquid culture was inoculated in the brain heart infusion (BHI) agar and incubated for 24 h to check the sterility of the positive culture tube [15]. To confirm MTBC, immunochromatography-based MPT64 rapid antigen detection kit (SD BIOLINE TB Ag, South Korea) was used [16]. The confirmed MTBC isolates were further subjected to DST using MGIT 960 system for ofloxacin (2ug/ml), kanamycin (2.5ug/ml), capreomycin (2.5ug/ml) and moxifloxacin (0.25ug/ml). The *H37Rv* strain was used as a quality control testing in DST.

### Statistical analysis

The sensitivity and specificity of GenoType MTBDR*sl* v.2.0 assay was analyzed with MedCalc statistical software taking the phenotypic DST as gold standard.

## Results

### GenoType MTBDR*sl* v.2.0 assay

Out of 1320 smear positive sputum samples, 1178 were identified as MTBC positive and 142 were negative by GenoType MTBDR*sl* v.2.0 assay. Of the 1178 valid results, 314 (26.6%) were sensitive to both FQs and SLIDs, 676 (57.3%) were only FQs resistant, and 188 (15.9%) were resistant to both FQs and SLIDs. Resistant mutations found in *gyrA* gene were the most prevalent (930), followed by *rrs* gene (172), *eis* gene (30) and *gyrB* gene (25). The distribution, as well as band pattern of mutations, is summarized in Additional file 1: Tables S1–S3.

### Drug susceptibility testing

Of the 264 samples inoculated for culture, 225 isolates were found MTBC positive in liquid culture, confirmed by immunochromatography-based MPT64 rapid antigen detection kit (SD BIOLINE TB Ag) with no growth on BHI agar plate, whereas 39 were found contaminated. Drug susceptibility testing of the 225 isolates by liquid culture showed that 39 (17.4%) were sensitive to both FQ (ofloxacin) and SLID (kanamycin), 138 (61.3%) were only FQ resistant and 48 (21.3%) were both resistant to FQs and SLIDs. The sensitivity and specificity of GenoType MTBDR*sl* v.2.0 assay in detecting resistance to FQs and SLIDs is described in Table 1.

**Table 1:** Performance of GenoType MTBDR*sl* v.2.0 assays compared Phenotypic DST in detecting resistance to FQs, SLIDs, and XDR-TB

**GenoType MTBDR*sl* v.2.0** **Assay**			**FQs**	
			**Phenotypic DST**	
	Resistant	Susceptible	Total	Sensitivity = 100%
Resistant	186	1	187	Specificity = 92.3%
Susceptible	0	38	38	NPV = 100%
Total	186	39	225	PPV = 98.41%
			SLIDs	
	Resistant	Susceptible	Total	Sensitivity = 100%
Resistant	48	0	48	Specificity = 100%
Susceptible	0	177	177	NPV = 100%
Total	48	177	225	PPV = 100%

			XDR-TB	
GenoType MTBDR*sl* v.2.0 Assay			Phenotypic DST	
	Resistant	Susceptible	Total	Sensitivity = 100%
Resistant	48	0	48	Specificity = 100%
Susceptible	0	177	177	NPV = 100%
Total	48	177	225	PPV = 100%

NPV = negative predictive value; PPV = positive predictive value, CI = 95%

### Concordance between GenoType MTBDR*sl* v.2.0 assay and Phenotypic DST

For assessing the performance of GenoType MTBDR*sl* v.2.0 assay, phenotypic DST was taken as gold standard. GenoType MTBDR*sl* v.2.0 assay accurately identified all strains except one, as shown in Table 2, thus the overall concordance between GenoType MTBDR*sl* v.2.0 assay and phenotypic DST was 224/225.

**Table 2:** Concordance between GenoType MTBDR*sl* v.2.0 Assay and phenotypic DST

Drugs	Concordant results		Discordant results	
	Resistant by both methods	Sensitive by both methods	Sensitive by phenotypic DST but resistant by GenoType MTBDR*sl* v.2.0 Assay	Resistant by phenotypic DST but sensitive by GenoType MTBDR*sl* v.2.0 Assay
FQs	186	38	1	0
SLIDs	48	177	0	0
XDR-TB	48	177	0	0

### Drug resistance pattern

The prevalence of overall resistance pattern to at least one drug was highest in ofloxacin 186/225, (82.6%), followed by kanamycin 48/225, (21.3%), capreomycin 46/225, (20.4%), and moxifloxacin 2/225, (0.88%) (Table 3).

**Table 3:** Resistance pattern to second-line anti-tuberculosis drugs among the 225 MTB isolates

Drugs	Total No. of drug resistant strains (%) N = 225	Total No. of drug susceptible strains (%)
Ofloxacin (2ug/ml)	186 (82.66)	39 (17.33)
Kanamycin (2.5ug/ml)	48 (21.33)	177 (78.66)
Capreomycin(2.5ug/ml)	46 (20.4)	179 (79.5)
Moxifloxacin(0.25ug/ml)	2 (0.88)	223 (99)

## Discussion

The present study highlights the rapid and accurate diagnosis of drug resistant-TB directly from sputum specimens by using GenoType MTBDR*sl* v.2.0 assay, whereas, previous studies have shown that it could be performed on DNA extracted from culture isolates [17–20]. According to WHO, an effective treatment regimen depends on optimal susceptibility testing of *M. tuberculosis* to anti-TB drugs [21].

Under TB control programs DR-TB portends a prominent threat globally. Efficient treatment of MDR-TB and XDR-TB is very expensive, which is especially an issue in low-income countries. Therefore, a sensitive and specific diagnostic tool is essential for effective diagnosis and subsequent treatment.

In our study, the specificity for FQs and SLIDs was 92.31% and 100% whereas sensitivity was 100% respectively by GenoType MTBDR*sl* v.2.0 assay in direct sputum samples when compared against phenotypic DST. A previous study by Tagliani et al. [21] demonstrated a sensitivity and specificity of GenoType MTBDR*sl* v.2.0 assay for FQs was respectively 93% and 98.3% and for SLIDs 88.9% and 91.7%, from culture positive isolates when compared with phenotypic DST.

A study from South Africa also showed that the GenoType MTBDR*sl* v.2.0 has shown an improvement in sensitivity and specificity for the determination of molecular resistance to both FQs (100% and 98.9%) and SLIDs (89.2% and 98.5%) compared with the pooled sensitivity and specificity of GenoType MTBDR*sl* v.1.0 as reported by the WHO Expert Group [12].

A study from Lithuania where the sensitivity and specificity of the GenoType MTBDR*sl* v.2.0 to detect ofloxacin resistance mutations in the *gyrA* and *gyrB* genes compared to DST was 91% and 100%. Whereas sensitivity and specificity to detect resistance mutations in the *rrs* and *eis* gene for aminoglycoside/cyclic peptides was 89% and 77% [22]. Brossier et al. [23] found the sensitivity and specificity of Genotype MTBDR*sl* v.2.0 assay was: FQs (94.8% and 98%), kanamycin (90.5% and 94.3%), amikacin (91.3% and 100%), capreomycin (83% and 100%), pre-XDR (83.3% and 88.6%) and XDR (83% and 100%), when compared with phenotypic DST. According to Yadav et al. [24], the sensitivity and specificity of Genotype MTBDR*sl* v.2.0 compared with phenotypic DST for detecting levofloxacin resistance was respectively 97.2% and 99.1%, and for kanamycin resistance was respectively 92.5% and 99.5%.

Further, in our study FQ resistance were mainly due to mutations in *gyrA*, indicated by both distribution as well as band patterns. The major mutation was reported in MUT3C (D94G, 20.12%), MUT1 (A90V, 2.54%), MUT3B (D94N/Y, 1.10%), MUT3A (D94A, 0.67%), MUT2 (S91P, 0.50%) and MUT3D (D94H, 0.25%). For SLID the band pattern of mutations in *rrs* gene was MUT1 (A1401G, 7.31%) and *eis* gene MUT1 (C-14T, 0.42%). In concordance with our result, Tagliani et al. [21] also reported the predominant mutations detected by Genotype MTBDR*sl* v2.0 as conferring FQs resistance in *gyrA* MUT1 (A90V, 35.6%) and MUT3C (D94G, 20.5%) mutations. According to data from Gardee et al. [12] the predominant mutation bands detected by MTBDR*sl* v2.0 conferring FQs resistance were in *gyrA* MUT3C (D94G, 44.2%), MUT3A (D94A, 13.5%), MUT3D (D64H, 11.5%) and MUT3B (D94N/Y, 9.6%), and the most frequently observed mutation for SLIDs resistance was *rrs* MUT1 (C1402T, 85.7%).

The present study also showed the drug-resistant profiles among 225 MTBC isolates by phenotypic DST, which were: 186 (82.6%), 48 (21.3%), 46 (20.4%) and 2 (0.88%) to ofloxacin, kanamycin, capreomycin and moxifloxacin, respectively. In a study from China, the phenotypic DST showed that among 170 M*. tuberculosis* isolates, 94 (55.3%), 25 (14.7%), 13 (7.6%) and 20 (11.8%) were resistant to ofloxacin, kanamycin, amikacin and capreomycin, respectively [25]. According to Yadav et al. [24], levofloxacin and kanamycin resistance using phenotypic DST was respectively, 176/415 (42.4%) and 40/415 (9.6%). A study from India showed phenotypic resistance to ofloxacin (59.1%), SLIDs (11.8%,) and to both antibiotics (10.0%) by phenotypic drug susceptibility testing using the MGIT 960 system [26]. According to Singh et al. [27], who used phenotypic drug susceptibility testing (DST) assess baseline resistance to second-line drugs DST, RR-TB positive patients also showed a high resistance to fluoroquinolones (FQs; levofloxacin 56%; moxifloxacin 44%) followed by kanamycin (8%) and capreomycin (6%). None of the patients were resistant to the other drugs tested (amikacin, clofazimine and linezolid).

## Conclusions

From this study we can clearly suggest that the sputum specimen with the clinical diagnosis of MTB could be directly subjected to GenoType MTBDR*sl* v.2.0 assay, after confirmation by GeneXpert MTB/RIF for rifampicin resistance (RR) and ZN staining. This bypasses the long incubation period required for liquid culture and DST and may help for the rapid detection of second line drug resistance for FQs and SLIDs in order to initiate treatment among the critical patients. The findings showed alarming level of FQ (ofloxacin) resistance from the geographical region (Uttar Pradesh) of India which contributes the highest cases of TB. However, another drug moxifloxacin could be used as treatment option owing to very low resistance.

## Supplementary Information

**Additional file 1: Table S1.** Band pattern of mutations in gyrA gene by GenoType MTBDR*sl* v.2.0 assay. **Table S2.** Band pattern of mutations in gyrB gene by GenoTypeMTBDR*sl* v.2.0 assay. **Table S3.** Band pattern of mutations in rrs gene by GenoTypeMTBDR*sl* v.2.0 assay. **Table S4.** Band pattern of mutations in eis gene by GenoTypeMTBDR*sl* v.2.0 assay.

## Data Availability

The datasets generated and/or analyzed during the current study are not publicly available due confidentiality agreement at the department of microbiology, Institute of Medical Sciences, Banaras Hindu University but are available from the corresponding author on reasonable request.

## Abbreviations

WHO: World Health Organization
DR-TB: Drug-resistant tuberculosis
FQs: Fluoroquinolones
SLIDs: Second-line injectable drugs
TB: Tuberculosis
MDR/RR-TB: Multi drug resistance/rifampicin resistance TB
MGIT: Mycobacterium growth indicator tube
MDR-TB: Multidrug-resistant TB
XDR-TB: Extensively drug-resistant TB
TAT: Turnaround time
LPAs: Line probe assays
MTBC: *Mycobacterium* *tuberculosis* *complex*
EMB: Ethambutol
DST: Drug susceptibility testing
RNTCP: Revised National Tuberculosis Control Programme
NALC-NaOH: *N*-Acetyl-l-cysteine and sodium hydroxide
AFB: Acid-fast bacilli
BHI: Brain heart infusion agar
