# Supplementary material for: Direct detection of resistance to fluoroquinolones/SLIDs in sputum specimen by GenoType MTBDRsl v.2.0 assay A study from Eastern Uttar Pradesh, India
Source: Ann Clin Microbiol Antimicrob. 2021 Aug 26;20:56. doi: 10.1186/s12941-021-00463-6 (PMC8394194; doi:10.1186/s12941-021-00463-6)
Supplement: Supplementary file 1 — Additional file 1: Table S1. Band pattern of mutations in gyrA gene by GenoType MTBDRsl v.2.0 assay. Table S2. Band pattern of mutations in gyrB gene by GenoTypeMTBDRsl v.2.0 assay. Table S3. Band pattern of mutations in rrs gene by GenoTypeMTBDRsl v.2.0 assay. Table S4. Band pattern of mutations in eis gene by GenoTypeMTBDRsl v.2.0 assay. [file 12941_2021_463_MOESM1_ESM.docx]

**Table S1.** Band pattern of mutations in gyrA gene by GenoType MTBDR*sl* v.2.0 assay

| **Gene** | **Wild type bands** | **Mut Bands** | | | | | | **No of isolates (%)** |
| --- | --- | --- | --- | --- | --- | --- | --- | --- |
|  |  | **gyrA MUT1** | **gyrA MUT2** | **gyrA MUT3A** | **gyrA MUT3B** | **gyrA MUT3C** | **gyrA MUT3D** |  |
| gyrA | WT1+WT2+WT3 | - | - | - | - | - | - | 733 (62.22) |
|  | WT1+WT2+WT3 | - | - | - | - | - | + | 3 (0.25) |
|  | WT1+WT2+WT3 | - | - | - | - | + | - | 6 (0.50) |
|  | WT1+WT2 | - | - | - | - | + | - | 223 (18.93) |
|  | WT1+WT2 | - | - | - | - | - | - | 15 (1.27) |
|  | WT1+WT2 | - | - | + | - | - | - | 7 (0.59) |
|  | WT1+WT2 | - | - | - | + | - | - | 12 (1.01) |
|  | WT1+WT2 | - | - | - | - | + | - | 4 (0.33) |
|  | WT1+WT2 | - | - | - | + | + | - | 1 (0.08) |
|  | WT1+WT2 | - | - | - | - | - | - | 4 (0.33) |
|  | WT1+WT3 | + | - | - | - | - | - | 29 (2.46) |
|  | WT1+WT3 | - | + | - | - | - | - | 6 (0.50) |
|  | WT1+WT3 | - | - | - | - | - | - | 8 (0.67) |
|  | WT2+WT3 | - | - | - | - | - | - | 2 (0.16) |
|  | WT1 | + | - | - | - | + | - | 1 (0.08) |
|  | WT1 | - | - | + | - | - | - | 1 (0.08) |
|  | WT1 | - | - | - | - | - | - | 4 (0.33) |
|  | WT2 | - | - | - | - | + | - | 2 (0.16) |
|  | WT2 | - | - | - | - | - | - | 1 (0.08) |
|  | WT3 | - | - | - | - | - | - | 4 (0.33) |
| gyrA | No Band | - | - | - | - | - | - | 16 (1.35) |
| gyrA | No Band | - | - | - | - | + | - | 1 (0.08) |
| No Band | No Band | - | - | - | - | - | - | 18 (1.52) |

**Table S2.** Band pattern of mutations in gyrB gene by GenoTypeMTBDR*sl* v.2.0 assay

| **Gene** | **Wild type bands** | **Mut Bands** | | **No of isolates (%)** |
| --- | --- | --- | --- | --- |
|  |  | **gyrB MUT1** | **gyrB MUT2** |  |
| gyrB | WT | - | - | 1093 (92.78) |
| gyrB | No band | - | - | 10 (0.84) |
| No band | No band | - | - | 11 (0.93) |

**Table S3.** Band pattern of mutations in rrs gene by GenoTypeMTBDR*sl* v.2.0 assay

| **Gene** | **Wild type bands** | **Mut Bands** | | **No of isolates (%)** |
| --- | --- | --- | --- | --- |
|  |  | **rrs**  **MUT1** | **rrs**  **MUT2** |  |
| rrs | WT1+WT2 | - | - | 1009 (85.65) |
|  | WT1+WT2 | + | + | 1 (0.08) |
|  | WT1+WT2 | + | - | 4 (0.33) |
|  | WT1+WT2 | - | + | 1 (0.08) |
|  | WT2 | + | - | 74 (6.28) |
|  | WT1 | - | - | 1 (0.08) |
| No Band | No Band | + | - | 8 (0.67) |
| No Band | No Band | - | - | 4 (0.33) |

**Table S4.** Band pattern of mutations in eis gene by GenoTypeMTBDR*sl* v.2.0 assay

| **Gene** | **Wild type bands** | **Mut Bands** | **No of isolates (%)** |
| --- | --- | --- | --- |
|  |  | **eis MUT1** |  |
| eis | WT1+WT2+WT3 | - | 1058 (89.81) |
|  | WT1+WT2+WT3 | + | 2 (0.16) |
|  | WT1+WT3 | - | 15 (1.27) |
|  | WT1+WT3 | + | 3 (0.25) |
|  | WT2+WT3 | - | 3 (0.25) |
